# Passing Events and Topics

**Published:** 1921-11

**Authors:** 


### PASSING EVENTS AND TOPICS.

The latest addition to the growing number of clubs for nurses is the United Nursing Services Club, 34 Cavendish Square, W., which opened its doors last month. Those eligible for membership are past and present members of the Naval and Military, the Territorial, the Royal Air Force, the Ministry of Pensions, and the British Red Cross Nursing Services. The Chairman is Dame Ethel Becher, R.R.C. The entrance fee is £1 1s., and the annual subscription two and a-half guineas for town or one and a-half guineas for country members. The Cowdray Club for Professional Women in Mr. Asquith's old house in Cavendish Square, which Viscount and Viscountess Cowdray purchased for the College of Nursing, will, it is expected, be the next women's club to be opened. Here trained nurses will have preferential treatment as regards subscription and fees, and the larger the number of original nurse members the greater reduction will it be possible to make. In addition to a large number of bedrooms which can ultimately be provided, the Cowdray Club will have a dining-room to seat 100 people, and there will be an ample supply of bathrooms and even a gymnasium.

---

Still another club for nurses and V.A.D. members is in contemplation. The Joint Council of the British Red Cross and the Order of St. John has for some time been casting about for suitable premises for such a club in London, which it now announces will soon be opened. It also proposes shortly to open a convalescent home, and it has already given and equipped a holiday hostel for nurses and V.A.D. members at Folkestone. This home, called St. Helena's, was opened on November 1. In this work the Joint Council acts in conjunction with the United Services Fund, and together they administer funds remaining in the hands of the Navy and Army Canteen Board at the end of the war.

---

The finances of the Birmingham hospitals, and especially of the General Hospital, are well known to be in a critical condition, and the recent efforts to improve them have not been free from difficulties and, indeed, criticism. We turn, then, with interest to a recent statement made by Mr. A. H. Leaney, Secretary of the Birmingham General Hospital, to the "Birmingham Evening Despatch." Two wards, containing fifty beds, have lately been closed, to the dismay of the patients, of the governors, and of the authorities of the Medical School, which maintains a large entry of students. The estimated deficit at the end of this year is £20,000; nearly half of the hospital's available securities have been sold in an attempt to pay off the debt; last year the income was £63,000 and the expenditure as high as £93,000.

---

We are living, said Mr. Leaney in the statement mentioned, in a new world, and the administration of the voluntary hospitals will have to be reorganised. For the next five years, he went on, it would be impossible for the income of the hospital to meet its expenditure. There is, then, little immediate chance of reopening the closed beds. But the board, it will be remembered, has started a scheme to increase the revenue. It has formed a General Hospital League, mainly to raise funds for the General Hospital. This League, which has numerous, if still insufficient, branches, is entirely apart from the workpeople's collection. It consists of a body of persons banded to arouse interest in the hospital among a class of persons hitherto not actively engaged in its support. So far, the Handsworth branch has succeeded in endowing one bed, and it is hoped that branches in other suburbs will follow suit. An effort is being made to reopen twelve of the fifty beds now closed, but everything depends upon the growth of the League.

---

Miss Stansfeld, in her address to the annual conference of the Chartered Society of Massage and Medical Gymnastics, sounded a note of caution against the indulgence in strenuous games or muscular exercises by the woman mentally exhausted by her professional work. Whilst emphasising the necessity for some form of exercise, and recommending certain movements which were effectively demonstrated by some of her senior pupils from Bedford, she frankly recognised that she did not expect one member of her audience to practise them. These movements, which could readily be done at home, were especially designed to increase circulation, respiration, and all the functions of life, and to strengthen the abdominal and other muscles. Miss Stansfeld, in addition to these trunk movements, recommended dancing, of the ballroom, Morris, and country and Greek types; walking and skipping; and even, where all other opportunities for exercise failed, the mounting and descent of tube staircases.

---

In his address at the same conference on "The Physiological Significance of Rest," Dr. Crichton Miller contrasted the sleep of man and animals, that of the latter dependent on security or insecurity, and being more superficial than the resting-time of a human being, because the animal's security from external danger is less. He referred to the connection between man's emotional life and his muscular state, and touched in an interesting and suggestive manner on the conflicts and repressions which are constantly waging in the human being, and with the methods of escape which are consciously, or perhaps unconsciously, employed. Such conflicts, intrinsically unescapable, are pressed downwards from the surface of the daily mental life, till an extraordinary capacity for deceiving ourselves is acquired. In dealing with the patients who cannot relax or cannot rest it is imperative to get at primary causes, and to get them to face conscious mental facts.

**PASSING EVENTS AND TOPICS—(*cont.*).**

Sir Alfred Mond's statement in the House of Commons, in reply to questions by Mr. Leonard Lyle, that King Edward's Fund, which is acting as the local committee for London of the Voluntary Hospitals Commission, has made considerable progress with its work, has been followed within a few days by the distribution of emergency grants to the most necessitous of their number (see p. 54). The Minister hopes that the personnel of a number of the provincial local committees will be complete within the next few weeks. He stated that it is not anticipated that the full amount of the grant will be spent during the current financial year, and an estimate will be submitted to the House next Session for a re-vote of the unexpended balance.

---

In our October issue we expressed the hope that the cry for economy at any price might not result in the hampering of the valuable work of the health services. We are glad to see that at least one economy has been abandoned, the Minister of Health having stated, in reply to several questioners in the House of Commons, that the fifty per cent. grant for milk for mothers and young children will be continued until the end of the present financial year. Sir Alfred Mond gave as his reasons for the continuation of the grant the commitments of local authorities and the distress arising from the extent of unemployment.

---

A lay paper rarely makes a careful criticism of hospital affairs, but when it does it deserves quoting. A valuable criticism was lately proffered by the "Derby Daily Telegraph" when commenting upon the decision of the Governors of the Derby Royal Infirmary to enlarge the weekly board from twenty-four to forty. The motive is praiseworthy, but we share the newspaper's hope that "the enlargement may not result in any decline of management efficiency, though the prevailing experience *probably* inclines to smaller rather than larger bodies for the effective discharge of duties of this kind." The adverb printed by us in italics is too timid: "certainly" is the proper word. But the remedy ought to lie not necessarily with a small committee, but with the self-control and self-effacement of the members.

---

In fixing the minimum standard for an approved hospital for existing nurses as fifty beds, with a daily average of not less than thirty-five occupied beds, the General Nursing Council for England and Wales has not aimed unduly high. It should be borne in mind that this standard, however, applies only to existing nurses, i.e. those trained before November 1919, and that the necessity for these small hospitals to affiliate with a larger institution under some scheme yet to be evolved, whereby the training of future nurses can be completely and satisfactorily carried out, is as acute as ever. We hope that the authorities of each small and special hospital will bend their mind to this problem, through the solution of which the difficulty of the supply of probationers and nurses may be overcome and the nurse's educational opportunities adequately safeguarded. The Rule drafted for future nurses by the Education and Examination Committee provides that the prescribed training which a nurse, applying for Registration on the general part of the Register after July 1924, shall have undergone is the training laid down in the Council's syllabus, and the fact that she has passed the examination of the Council upon that training shall be evidence that she possesses the prescribed training.

---

The Committee of the Royal Gwent Hospital, Newport, has provisionally accepted a tender for the building of a new dispensary and out-patient department. The cost will be about £38,000, and the new buildings will form part of the general extension scheme. The tender, supplied by Mr. Moon, anticipates that the work will take about two years to complete. The decision represents an important step forward in the growth of this hospital, which has a big future and already a name of its own, thanks largely to the support won by its directors.

---

At the recent annual meeting of the West Bromwich District Hospital a suggestion was made that in the near future it might be possible to give some honorarium to the medical staff for their services. This suggestion has been very strongly negatived by the medical staff themselves, who hold to the principle that all the medical work should be honorary.

---

Mr. J. F. Royce, the former Secretary of Harrogate Infirmary, has taken advantage of the jubilee of this hospital to publish a sketch of its history in the "Ripon Gazette." On March 31, 1870, it was resolved to found a cottage hospital, and premises were taken in Belford Row, Tower Street, where two cottages were knocked into one. In the following September the first two patients were admitted, and the public opening took place. There were six beds in charge of an honorary medical officer and a "nurse-housekeeper," and the first year ended with a balance in hand of about £150. In 1873 the first move took place to larger premises in Avenue Road and Tower Street. In 1883 the site of the present buildings was acquired, and in 1902 it changed its title, with its size, to that of the Harrogate Infirmary. Mr. Richard Ellis was Chairman from 1871 to 1895, and the presidency is still in the Greenwood family. A new wing was added in 1906, and the time is now ripe for another enlargement, and an adjoining site has been already purchased. It was not till 1909 that a resident house surgeon was appointed. Hitherto the medical work was voluntary, a fact which Mr. Royce records with pride and gratitude in his valuable sketch. If he would expand his work, and become in full the historian of the hospital, he would crown his services in the most appropriate way, for he has made this field his own.

**PASSING EVENTS AND TOPICS—(*cont.*).**

The hospitals at Altrincham, Birkenhead, Chester, Crewe, Ellesmere Port, Hoylake and West Kirby, Macclesfield, Northwich, Runcorn, Stockport, Tarporley, Wallasey, and Winsford were represented at a meeting of the voluntary hospitals of Cheshire held at the Chester Royal Infirmary last month. The meeting considered the Voluntary Hospitals Committee's final report, recently published, and the representation of voluntary hospitals on local committees. It also discussed scales of payments by public authorities, waiting admission lists, appeals, patients' payments, grants from Approved Societies, insurance companies, and hospitals, and grants for nurse-training.

---

Personal responsibility for the transmission of venereal disease has been upheld by both civil and criminal courts in America. In Oklahoma a man has been sentenced to five years in the penitentiary for infecting a girl with syphilis. In Nebraska the court upheld a doctor who warned an hotel-keeper that one of his patients, a guest at the hotel, had syphilis, and had refused treatment and was consequently a menace to the public health. In North Carolina a woman has been awarded $10,000 damages against her husband for a similar infection, and the Supreme Court upheld the judgment.

---

The divorce of public health work from that of the school medical service is a matter for regret, both on rational and economic grounds. Dr. Charles Porter, the medical officer of health for the Metropolitan borough of St. Marylebone, in his annual report for 1920, writes the following trenchant criticism of this duplication of authorities who are both engaged in the prevention of disease:

The Medical Officer of Health of a borough in the Metropolis is not school medical officer. The work in connection with both schools and school children is entirely in the hands of the London County Council and their officers, the local medical officer of health having little or nothing to do with it. In no part of the kingdom, indeed, is the divorce between the general public health work and school medical work so complete as in the Metropolis, and since it is impossible to see any justification for it on public health grounds, certainly, and probably also on economic grounds, it is difficult to see why either the central government authorities or the local authorities have for so long acquiesced in the arrangement.

For the reason that this question of reorganisation of public health in London is under consideration and this as well as other anomalies are or will soon be under discussion, it is not proposed to take it up here. It may be stated, however, that because of the existence of the two authorities there is frequently duplication of work, a quite unnecessary and excessive amount of interchange of correspondence and from time to time cross-purposes. All of which is contrary to the best interests of the public health locally and particularly to those of the children.

In regard to the infectious diseases, as they affect the school child, that there should be two staffs—the local and the County Council—engaged, appears to be unnecessary and generally leads merely to the expenditure of a great deal of energy as a result of each endeavouring to keep the other informed as to the state of affairs.

That, so far as the general health of the school children, as determined by medical inspection, is concerned, only one authority, and that not the local authority but the County Council, possesses full information, is a serious matter. In regard to conditions found at the medical inspections, the local medical officer of health receives no direct information; as a matter of fact, if he desires to learn where inspections are to be made he must ask to be informed or find out from the weekly gazette of the County Council. Again, if he wishes to know what the results were, he must ask or endeavour to glean the information from the pages of reports issued by the County Council long afterwards.

These are, clearly, serious objections, and it is sincerely to be hoped that when reorganisation takes place school medical work will be put in the hands of the local health authority, the Borough Council.

---

The importance of the administration of anti-tetanic serum at the time of injury in all cases of lacerated and contused wounds has long been recognised, and the efficiency of this measure in reducing the incidence of tetanus was shown in a most convincing manner in the recent war. Twelve months ago the Department of Public Health in New Zealand arranged that every hospital board in the Dominion should be supplied with stocks of anti-tetanic serum, to be issued free for use in necessitous cases, and at cost price otherwise. At the same time, all medical practitioners were communicated with in a circular memorandum notifying them of the arrangement, and pointing out the desirability of their making routine use of this serum as a prophylactic in the case of any injuries where direct or indirect contamination with earth, manure, etc., had occurred.

---

The following statement has been circulated by the Government Staff News Association:

Criticisms having been made with respect to the alleged large number of medical men in full-time employment by Government Departments, the appended authoritative statement may be of interest with regard to the Ministry of Pensions. The Ministry of Pensions employs 297 whole-time medical officers, who are engaged solely in administering treatment to ex-Service men at our hospitals and clinics. They have under their care about 11,000 in-patients and 106,000 out-patients at the present date. The services of a considerable number of part-time and consulting medical officers are also utilised.

The Ministry also employs on administrative work 316 full-time medical officers, of whom fourteen are stationed at headquarters in London. These officers are in general control of the medical side of the work of the Ministry and advise the Minister on questions of medical policy. The remainder are stationed in the various districts into which the United Kingdom is divided for administrative purposes, and of these more than one-third of the total are the medical officers in charge of these districts. Of the remainder some are engaged mainly in supervising and co-ordinating the work of medical boards, and in providing for the latter the appropriate medical *personnel*. Others are specialists, who are responsible for supervising and controlling the treatment of pensioners suffering from various disabilities which call for special training and experience, such as neurasthenia, nerve injuries, and tropical diseases. A further number are engaged in work connected with the assessment of the degree of disablement and on work in connection with general treatment, domiciliary visits to bed-ridden pensioners, inspection of hospitals and clinics, medical appeal boards, etc.

At present there are 1,150,000 ex-Service men and women in receipt of pensions, and the number of full-time

**PASSING EVENTS AND TOPICS—(*cont.*).**

doctors hardly seems excessive, though with the introduction of permanent pensions under the new Act it is hoped that the medical work will be reduced considerably.

Those of us who have attempted to cleanse the head of a verminous child know how difficult it is to remove all the nits. Sometimes, indeed, the only real cure is to cut the hair short. This practice may, in certain instances, be desirable for the moral effect that it produces on the friends and neighbours of the infested child; but the cutting of hair to remedy infestation is not universally a desirable measure, and is calculated to do more harm than good to the popularity of the school medical service. There is a comb now on the market, by the aid of which it is not difficult to remove nits from a child's hair. The comb is strongly made, and was certainly efficient in removing nits from the hair of a girl on whom we used it. The comb is manufactured by J. Sacker, 13 Blackstock Road, Finsbury Park, London, N. 4.

---

Public health work is evolving at so rapid a rate that one hesitates to say to-day what will be in vogue to-morrow. The lesson of the war, with its physical examinations of drafted men, has taken so great a hold upon the people of the country that everyone has become a potential health officer. Public opinion has been stirred to its very foundations in regard to health and the prevention of disease and disability. From the White House to the lowest hovel, people are thinking in terms of public health and are on tiptoe to do something which will benefit the community, the State, and the nation.

(U.S.A. Public Health Reports.)

---

Typhus is not the only menace from Russia. It requires but little expert knowledge of epidemiology to recognise that when a community is underfed and its general resistance lowered, when clothing is scanty and difficult to procure, and when the supply of soap and other material for cleansing purposes is exhausted, typhus, cholera, enteric fever, variola, and other epidemic diseases will get out of control. Experience teaches, in addition, that syphilis becomes, under such conditions, a national scourge, widespread and immensely virulent. It is therefore not sufficient for the safety of the whole of Europe to attack typhus in Poland and Galicia. Relief must be extended to the starving and shivering people of central Europe generally, so that the main ætiological factor, common to these epidemics, may be removed.—"Medical Journal of Australia."

---

**Registration of Nurses.**

We are asked to remind applicants for registration that requests for application-forms should be accompanied by a long stamped addressed envelope. The applicant should state (a) for which part of the Register she is eligible, i.e., for the General Part, or the Supplementary Parts for Sick Children's Nurses, Mental Nurses, Nurses for Mental Defectives, or Fever Nurses, and (b) whether as an "Existing Nurse" or an "Intermediate Nurse." The former means a nurse who completed her training before November 1, 1919 (when the Nurses' Registration Act became law), and the fee is one guinea. The latter is one who completed her training after November 1, 1919, and the fee is two guineas.

Applications should be addressed to The Registrar, General Nursing Council for England and Wales, 12 York Gate, Regent's Park, London, N.W. 1.

### EMERGENCY GRANTS TO LONDON HOSPITALS.

**Statement by the Voluntary Hospitals Commission.**

The King Edward's Hospital Fund, as the Local Voluntary Hospital Committee for London, have now reported to the Hospitals Commission in regard to the situation of the London hospitals. They estimate that the aggregate deficit on the maintenance account for the year ending December 31, 1921, will amount to at least £360,000. This is less than the corresponding figure for 1920, but the gravity of the situation is shown by the fact that without exception all the larger general hospitals, including all the medical schools, will show heavy deficits. Moreover, a number of hospitals have now exhausted all their realisable assets, and without immediate assistance will have no alternative but to close beds. It is a condition of the Government grant of £500,000, which has to meet the needs not of London only but of the whole of Great Britain, that a corresponding amount must be raised by the hospitals themselves. As the total deficits for the year were estimated by Lord Cave's Committee at £1,000,000, the importance of the pound for pound rule is manifest.

The Hospitals Commission have provisionally appropriated £180,000 for London, which is half the total deficits for the year as estimated by the King's Fund. But if the London hospitals are to earn this grant they must themselves raise a similar amount. In view, however, of the fact that a number of hospitals have already, or shortly will have, exhausted their realisable assets, the Commission have decided in certain cases to make emergency grants in anticipation of the new money which will have to be raised. These grants are in no case more than half the amount of the estimated deficits for 1921; and the Commission have not felt justified at this stage in making emergency grants to any hospitals whose realisable assets exceed their liabilities, as such hospitals not having exhausted their credit are not compelled to close beds immediately, but can continue their activities at any rate for the present.

The total amount of the present emergency grant is £77,900, which is to be distributed as follows:—

Canning Town, £700; East London Hospital for Children, £3,000; Elizabeth Garrett Anderson Hospital for Women, £600; Great Northern Central Hospital, £11,000; Hampstead General Hospital, £3,400; Hospital for Epilepsy, £850; Hospital for Sick Children, £4,000; Infants' Hospital, £1,000; King's College Hospital, £9,000; London Temperance Hospital, £2,000; Metropolitan Hospital, £750; Middlesex Hospital, £3,000; Mildmay Mission Hospital, £400; National Hospital for the Paralysed, £800; Prince of Wales's General Hospital, £2,200; Queen Charlotte's Lying-in Hospital, £3,000; Queen Mary's Hospital for the East End, £1,000; Queen's Hospital for Children, £2,100; Royal Free Hospital, £12,000; Royal National Orthopædic Hospital, £3,000; Royal Waterloo Hospital for Women and Children, £500; Samaritan Free Hospital for Women, £800; South London Hospital for Women, £800; University College Hospital, £12,000—total, £77,900.

